# Secreted frizzled related protein 1 protects H9C2 cells from hypoxia/re-oxygenation injury by blocking the Wnt signaling pathway

**DOI:** 10.1186/s12944-016-0240-5

**Published:** 2016-04-06

**Authors:** Jing Tao, Mayila Abudoukelimu, Yi-tong Ma, Yi-ning Yang, Xiao-mei Li, Bang-dang Chen, Fen Liu, Chun-hui He, Hua-yin Li

**Affiliations:** Department of Cardiology, the First Affiliated Hospital of Xinjiang Medical University, Li Yu Shan South Road 137, Urumqi, 830001 People’s Republic of China; Xinjiang Key Laboratory of Cardiovascular Disease Research, Li Yu Shan South Road 137, Urumqi, 830001 People’s Republic of China; Xinjiang Medical University, Li Yu Shan South Road 137, Urumqi, 830001 People’s Republic of China

**Keywords:** H9C2, Sfrp1, Wnt signaling pathway, Apoptosis, Hypoxia reoxygenation injury

## Abstract

**Background:**

In animal models, secreted frizzled related protein 1 (Sfrp1) inhibition of the Wnt signaling pathway is beneficial because Sfrp1 reduces myocardial apoptosis and prevents heart failure. The mechanisms mediating the cellular survival effect of Sfrp1 has not been completely elucidated. The present study was designed to investigate the possible protective actions of Sfrp1 on cardiac muscle cells using an in vitro model of ischemia/reperfusion, and to evaluate the possible involvement of the Wnt signaling pathway.

**Methods:**

We used a recombinant AAV9 vector to deliver the Sfrp1 gene into H9C2 rat cardiomyoblasts and adopted an in vitro model of ischemia/reperfusion. Cell vitality was measured by CKK-8 and the trypan blue exclusion assay. Western blot was used to evaluate the expression of Dvl-1, β-catenin, c-Myc, Bax, and Bcl-2. Flow cytometry analysis of cardiomyocyte apoptosis was performed.

**Results:**

We confirmed that Sfrp1 significantly increased cell viability (assayed by trypan blue and CKK-8) and decreased apoptosis (assayed by flow cytometry analysis and the Bax/Bcl-2 ratio). These effects were partly attributable to the ability of Sfrp1 to down-regulate Wnt signaling pathway (assayed by Western blot to evaluate the expression of Dvl-1, β-catenin, and c-Myc). Indeed, reactivation of the Wnt signaling pathway activity with the specific activator, Licl, reduced Sfrp1-induced cardioprotection during hypoxia and reoxygenation.

**Conclusions:**

The present study demonstrated that Sfrp1 directly protected H9C2 cells from hypoxia and reoxygenation-induced reperfusion injury and apoptosis through inhibition of the Wnt signaling pathway, and added new mechanistic insight regarding the cardioprotective role of Sfrp1 on ischemic damage.

**Electronic supplementary material:**

The online version of this article (doi:10.1186/s12944-016-0240-5) contains supplementary material, which is available to authorized users.

## Background

Myocardial infarction remains a significant and unsolved health problem that seriously affects human health worldwide [1, 2]. One of the most significant factors responsible for the high mortality and poor recovery rate of myocardial infarction is myocardial ischemia/reperfusion (I/R). It is well-known that apoptosis is a significant cellular mechanism responsible for I/R injury in the myocardium. Exploring anti-apoptotic agents is a novel therapeutic option to prevent or reduce I/R-induced myocardial injury [3–5].

In this context, secreted frizzled related protein 1(Sfrp1) emerges as a feasible therapeutic candidate. Sfrp1 has been shown to be beneficial in improving cardiac structure and function post-myocardial infarction (MI) in rodents [6]. In addition, Sfrp1 has been shown to be important for vascular cell proliferation in vitro and in vivo [7]. Of the five secreted frizzled related proteins (Sfrps), Sfrp1 is present during both cardiac development and adult life, and is abundantly expressed in the mouse and human heart [8]. The mechanism underlying the cellular survival effect of Sfrp1 has not been completely elucidated. We have recently demonstrated that Sfrp1 exerts protective effects against H_2_O_2_-induced apoptosis in rat cardiomyocytes [9]; however, the underlying mechanism is still unclear. It is currently thought that Sfrp1 competes with the frizzled receptor for Wnt ligands, thereby preventing the activation of Wnt signaling [10]. Through this interaction, Sfrp1 could potentially influence cell fate and survival. Accordingly, we hypothesized that Sfrp1 exerts an anti-apoptotic effect by antagonizing Wnt signaling with pro-apoptotic properties.

The present study was designed to investigate the possible protective actions of Sfrp1 on cardiac muscle cells using an in vitro model of I/R, i.e., hypoxia and reoxygenation (H+R), and to evaluate the possible involvement of the Wnt signaling pathway.

## Methods

### Cell culture

H9C2 embryonic rat myocardium-derived cells, a well-characterized and widely used cell line to study myocardial cell ischemia [11], were obtained from China Center for Type Culture Collection ([CCTCC], China). The cells were cultured in DMEM (Gibco, USA) supplemented with 10 % heat-inactivated fetal bovine serum (FBS; Gibco, USA), 2 mM glutamine(Sigma, USA), and 2 mM penicillin-streptomycin (HyClone, USA) in a 5 % CO_2_ humidified atmosphere at 37 °C.

### I/R injury model in vitro and experimental protocols

H9C2 cells were subjected to H+R and simulated in vitro by substrate starvation plus hypoxia and reoxygenation, as previously described [12]. The cells were incubated in DMEM with no serum or glucose and placed in a hypoxic chamber saturated with a 0.1 % O_2_, 5 % CO_2_, and approximately 95 % N_2_ gaseous mix, humidified, and warmed at 37 °C for 7 h. At end-hypoxia, the cells were reoxygenated for 2 h by incubation under normoxic conditions in glucose-containing, serum-free DMEM. Control normoxic cultures were also prepared. H9C2 cells with or without recombinant AAV9 vector transfection containing the Sfrp1 gene (AAV9-Sfrp1) were kindly provided by Virovek (Hayward, USA). The multiplicities of infection (MOI) of AAV9-Sfrp1 were selected from preliminary experiments (MOI = 6 × 10^5^vg/cell), and demonstrated that infection efficiency peaked on the fifth day of transfection [9]. AAV9-Sfrp1 was added preventatively as follows: cells were transfected with AAV9-Sfrp1 (MOI = 6 × 10^5^vg/cell), and subjected to H+R (AAV9-Sfrp1+ H+R) 5 days later. To investigate the role of the Wnt signaling pathway with respect to the mechanism of action of Sfrp1, H9C2 cells were treated with lithium chloride ([Licl] 20 mM; Sigma, USA), an activator of the Wnt signaling pathway, for 12 h on the 5th day of AAV9-Sfrp1 transfection.

### Reverse transcription-polymerase chain reaction (RT-PCR) assay

To evaluate whether or not H9c2 cells highly express Sfrp1 mRNA after AAV9-Sfrp1 transfection, 1 mg of total RNA extracted with TRIzol reagent (Invitrogen, USA), was reverse transcribed and amplified with the SuperScript One-StepRT-PCR system (Invitrogen, USA). After cDNA synthesis for 30 min at 55 °C, the samples were pre-denatured for 5 min at 95 °C,then subjected to 35 cycles of PCR performed at 95 °C for 30s, alternating with 59.6 °C for 30s and 72 °C for 45 s. The final extension step was performed at 72 °C for 10 min. The following rat gene-specific primers were designed using Primer 5.0 software based on the following reported rat sequences: Sfrp1, forward: 5′-ATGCAGTTCTTCGGCTTCTACT-3′ and reverse: 5′-CAGCTTCTTCAGCTCCTTCTTC-3′. PCR products were electrophoresed on a 3 % agarose gel stained with ethidium bromide.

### Cell counting kit-8 viability assay

A cell counting kit-8 (CCK-8; Dojindo, Japan) was used to measure the cell viability according to the manufacturer’s instructions. The H9C2 cells were seeded in 96-well plates at 1 × 10^4^cells/well. At the end of the treatment period, 10 μl of WST-8 solution was added to the cells and the cells were incubated for 2 h at 37 °C. The absorbance of each well at 450 nm related to the reference absorbance at 630 nm was measured using a microplate reader (Bio-Rad, USA). The cell viability percentage was calculated using the following formula: % cell viability = (mean absorbance in the test wells)/(mean absorbance in the control well) × 100.

### Trypan blue viability assay

The trypan blue exclusion method was used to further assess cell viability. H9C2 cells (5 × 10^4^per well) were seeded in 24-well plates. At the end of treatment, the cells were gently harvested and mixed with 0.4 % trypan blue solution (Sigma, USA). The resulting cell suspension was counted under a phase-contrast inverted microscope using a Burker chamber. The viable cells were expressed as a percentage of the total counted cells.

### Western blot analysis

After treatment, H9C2 cells were lysed in cold RIPA buffer (Thermo, USA), added with Halt Protease Inhibitor Cocktail (Thermo, USA). After centrifugation at 14,000 g for 15 min at 4 °C, the supernatants were collected, and the total protein content was measured spectrophotometrically using the BCA Protein Assay kit (Thermo, USA). Western blot analysis was performed according to standard procedures. Equal amounts of protein (40 μg) from each sample were separated on SDS-PAGE precast gels (Invitrogen, USA) and transferred to nitrocellulose membranes (Invitrogen, USA). After blocking with 5 % skim milk, the membranes were blotted with antibodies against β-catenin, c-Myc, Bax, Bcl-2, GADPH (all from Cell Signaling, USA) or Dvl (Santa, USA) using the Western Breeze chromogenic immunodetection system (Invitrogen, USA). The images were captured and quantified with Image Lab 4.0 software (Bio-Rad Laboratories), and the values were normalized to GAPDH.

### Flow cytometry analysis of cardiomyocyte apoptosis

Apoptosis was assessed using the Annexin V-FITC/PI Apoptosis Detection kit (KeyGEN, China) according to the manufacturer’s protocol. Cells were washed with ice-cold PBS, then re-suspended in binding buffer. A total volume of 5 μL of Annexin V-FITC stock solution was added and incubated in the dark for 15 min at room temperature. Immediately after mixing with 5 μL of propidium iodide solution for another 5 min in the dark, the apoptotic cells were identified by flow cytometry (Beckman Coulter, USA).

### Blind test

For the current study, the technicians were blind to the experimental group while running the tests and recording the data.

### Statistical analysis

Statistical analysis was performed with the SPSS (version 22.0; Chicago, IL) software package. The values for all measurements were presented as median and interquartile range. All assays were repeated at least three times in triplicate. Based on the limited sample size and non-normality of continuous values, post-hoc analyses of group differences were assessed with Kruskal-Wallis pairwise comparison. Statistical significance was set at the standard level (*p* <0.05), and the Bonferroni correction was applied in post-hoc analyses (α = 0.05/6 = 0.0083).

## Results

### Sfrp1 gene is expressed in H9C2 cells

**Fig. 1:**
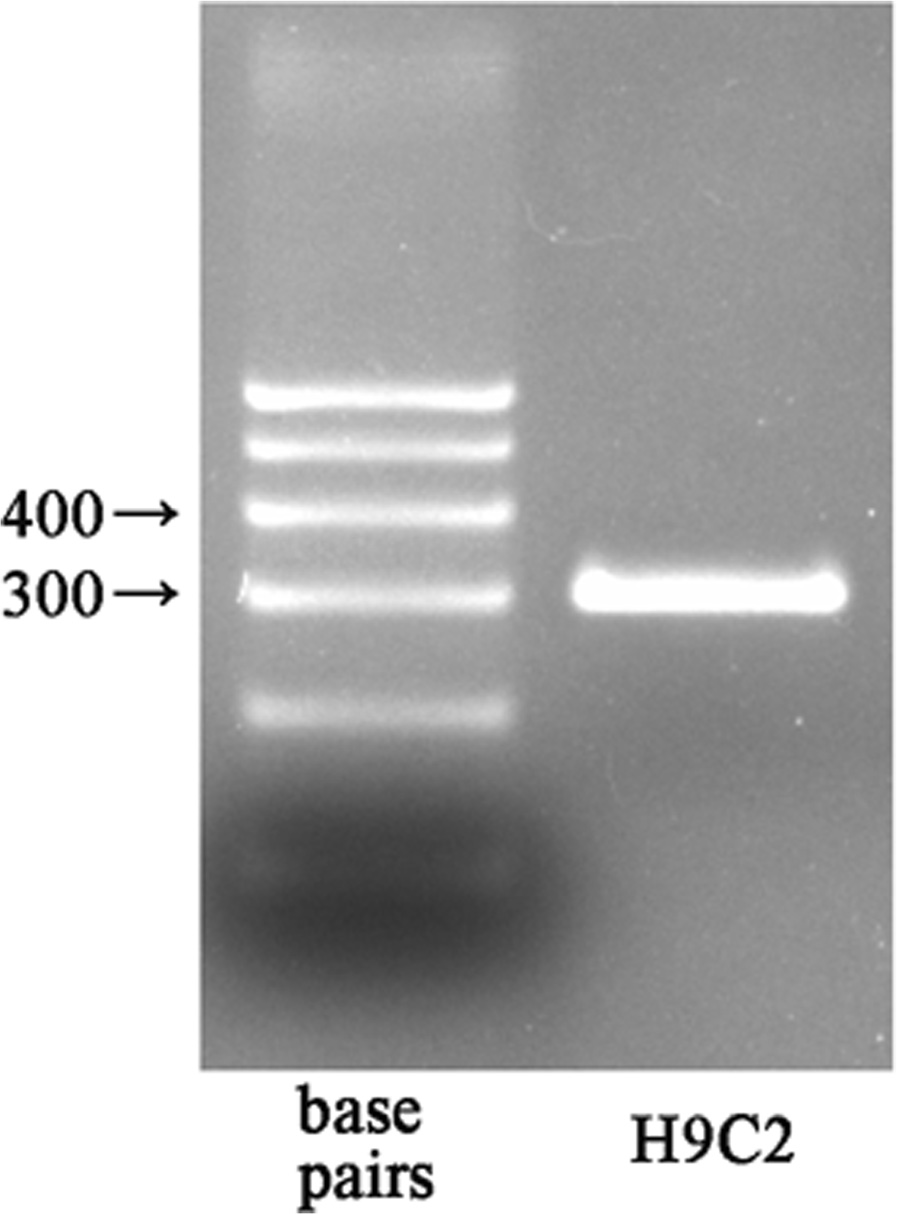
RT-PCR showing Sfrp1 mRNA in H9C2 cells

The present findings showed that H9C2 cells highly expressed Sfrp1 mRNA after AAV9-Sfrp1 (MOI = 6 × 10^5^vg/cell) 5 days after transfection (Fig. 1).

### Sfrp1 increases H9C2 cell viability impaired by H+R

**Fig. 2:**
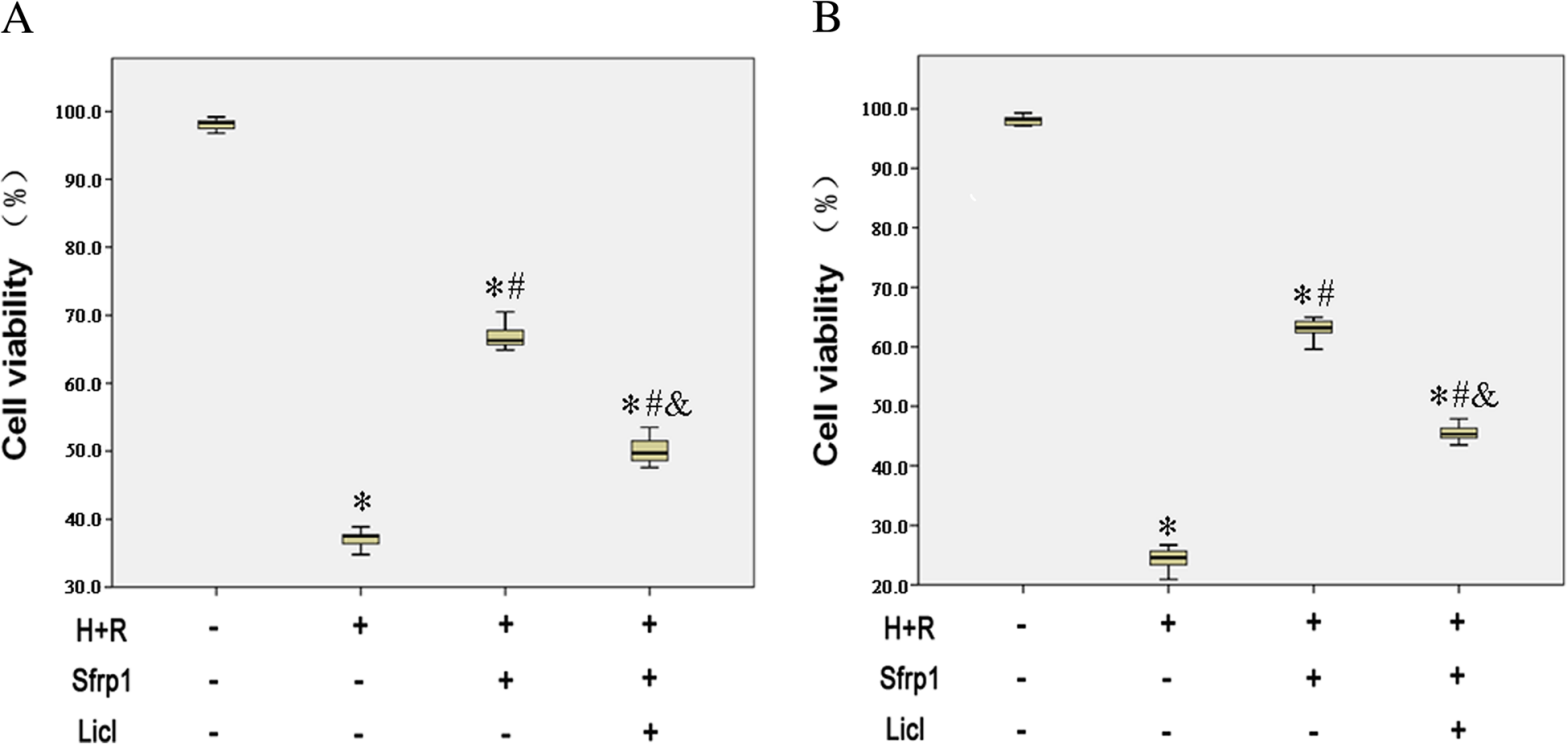
Evaluation of H9C2 cell viability by CKK-8 (**a**) and trypan blue exclusion (**b**) assay. Hypoxia and reoxygenation (H+R) cause a marked reduction in the number of viable cells. This effect was antagonized by Sfrp1-transfected cells before hypoxia and reoxygenation (Sfrp1+H+R). The cytoprotective effects of Sfrp1 were significantly reduced by the Wnt signaling pathway activator, Licl. Median values with interquartile range (*box*) and range (whiskers). Post-hoc analyses of group differences were assessed with Kruskal-Wallis pairwise comparison. Statistical significance was set at the standard level (*p* <0.05), and the Bonferroni correction was applied in post-hoc analyses (α = 0.05/6 = 0.0083). (Note:* vs. control *P* <0.001; # vs. H+R *P* <0.001; vs. Sfrp1+H+R *P* <0.001)

The CKK-8 assay showed that H+R caused a marked reduction in H9C2 cell viability [37.5 (36.0, 38.2) versus 98.3 (97.4, 98.9) %; *p* <0.001] compared to control group. AAV9-Sfrp1-transfected cells before H+R significantly increased cell viability after H+R [66.3 (65.5, 69.0) %; compared with other groups all *P* <0.001]. The beneficial effects of Sfrp1 transfection were significantly reduced when the Wnt signaling pathway activator, Licl, was administered together [49.7 (48.6, 52.4) %], indicating that the Wnt signaling pathway is involved in the cardioprotective role of Sfrp1 against cardiac injury (Fig. 2a).

Similar findings were obtained with the trypan blue exclusion test (Fig. 2b), which showed that H+R caused a marked reduction of H9C2 cell viability [24.6 (22.5, 25.8) versus 98.2 (97.3, 98.8) %; *p* <0.001] compared to control group. AAV9-Sfrp-transfected cells before H+R significantly increased cell viability after H+R [63.2 (62.4, 64.4) %; compared with other groups all *P* <0.001]. The effects of Sfrp1 were reduced by co-administration of Licl [45.3 (44.3, 46.6) %].

### Sfrp1 protects H9C2 cells from apoptosis-induced by H+R

**Fig. 3:**
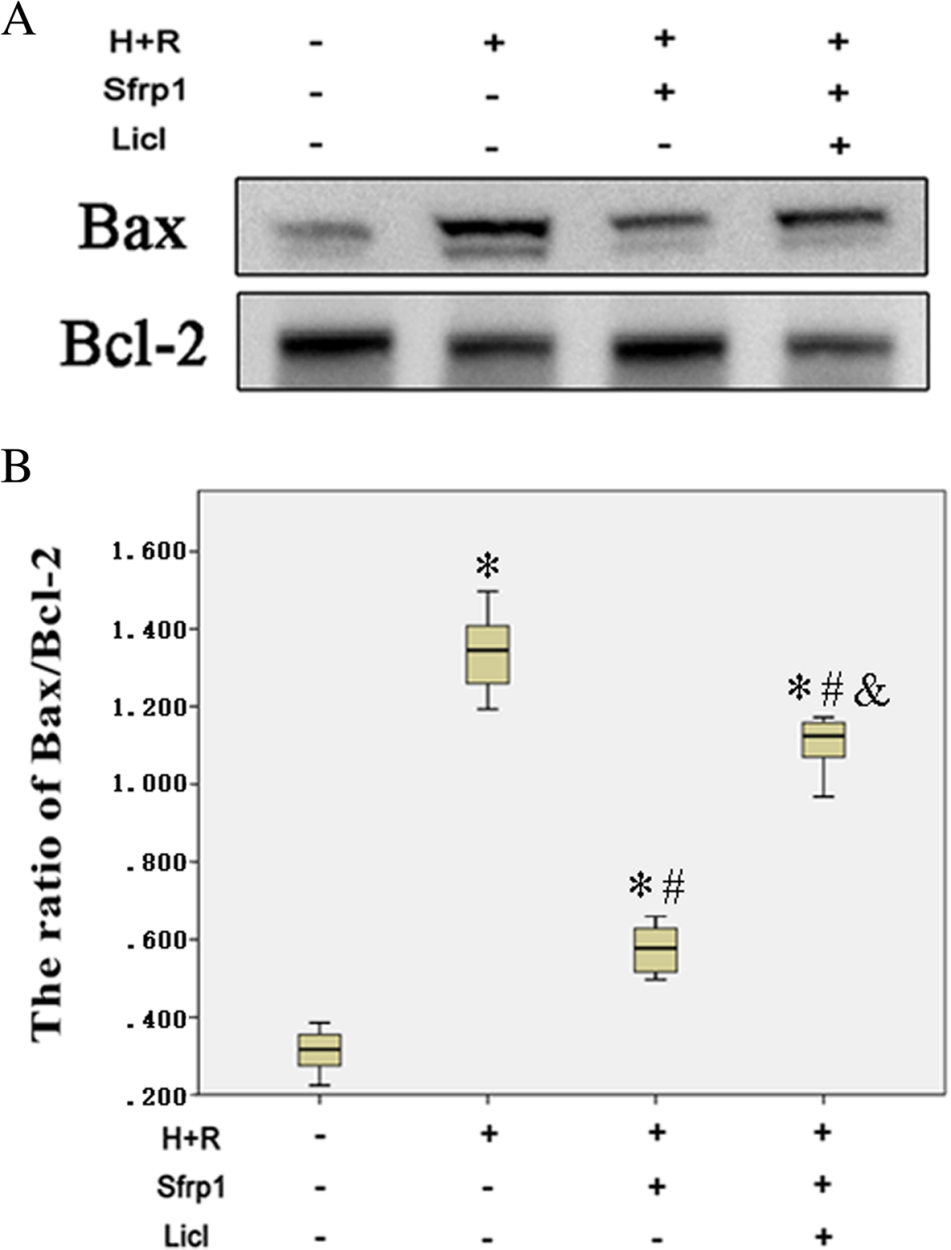
**a** Western blot shows that the expression of the anti-apoptotic protein, Bcl2 was reduced and Bax was enhanced by hypoxia and reoxygenation (H+R). These changes were antagonized by Sfrp1-transfected cells before hypoxia and reoxygenation (Sfrp1+H+R). The Wnt signaling pathway activator, Licl, reduced the effects of Sfrp1. **b** Median values with interquartile range (*box*) and range (whiskers). Post-hoc analyses of group differences were assessed with Kruskal-Wallis pairwise comparison. Statistical significance was set at the standard level (*p*<0.05), and the Bonferroni correction was applied in post-hoc analyses (α=0.05/6=0.0083). (Note:* vs. control *P*<0.001; # vs. H+R *P*<0.001; vs. Sfrp1+H+R *P*<0.001)

Sfrp1 significantly decreased apoptotic death induced by H+R in H9C2 cells (Fig. 3). Indeed, compared with controls, expression of the anti-apoptotic protein, Bcl2, was reduced and the pro-apoptotic protein, Bax, was enhanced by H+R. AAV9-Sfrp1 transfection before H+R increased the expression of Bcl2 and decreased the expression of Bax. The effects of Sfrp1 were reduced by co-administration of Licl.

**Fig. 4:**
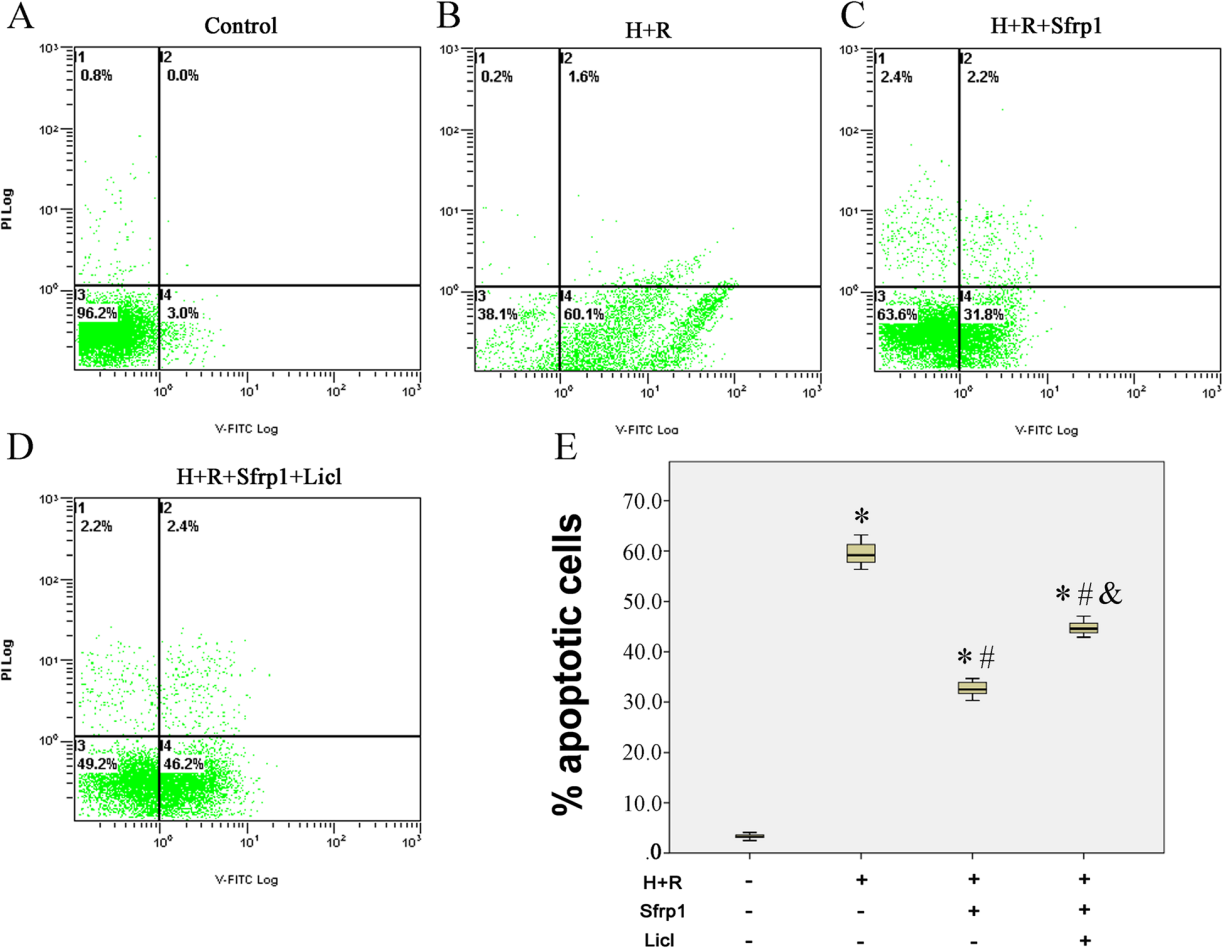
AnnexinV-FITC/PI double staining flow cytometry to detect the apoptosis rate. The apoptosis rates of H9C2 cells were increased by hypoxia and reoxygenation (H+R). These changes were antagonized by Sfrp1-transfected cells before hypoxia and reoxygenation (Sfrp1+H+R). The Wnt signaling pathway activator, Licl, reduced the effects of Sfrp1. **a** Control group. **b** H+R group. **c** Sfrp1+H+R group. **d** Sfrp1+H+R+Licl group. **e** Median values with interquartile range (*box*) and range (whiskers). Post-hoc analyses of group differences were assessed with Kruskal-Wallis pairwise comparison. Statistical significance was set at the standard level (*p*<0.05), and the Bonferroni correction was applied in post-hoc analyses (α=0.05/6=0.0083). (Note:* vs. control *P*<0.001; # vs. H+R *P*<0.001; vs. Sfrp1+H+R *P*<0.001) (Additional files 1, 2, 3 and 4)

Evaluation of the apoptosis rates, as measured by the flow cytometry method, was consistent with these findings (Fig. 4). H+R caused a marked increase in apoptosis [59.2 (57.6, 61.7) versus 3.2 (2.9, 3.7) %; *p* <0.001] compared to control group. AAV9-Sfrp1 transfection before H+R significantly decreased apoptosis after H+R [32.5 (31.5, 34.3) %; compared with other groups all *P* <0.001]. As expected, co-administration of Licl reduced the effects of Sfrp1 [44.6 (43.5, 46.1) %].

### Sfrp1 inhibits the Wnt signaling pathway

**Fig. 5:**
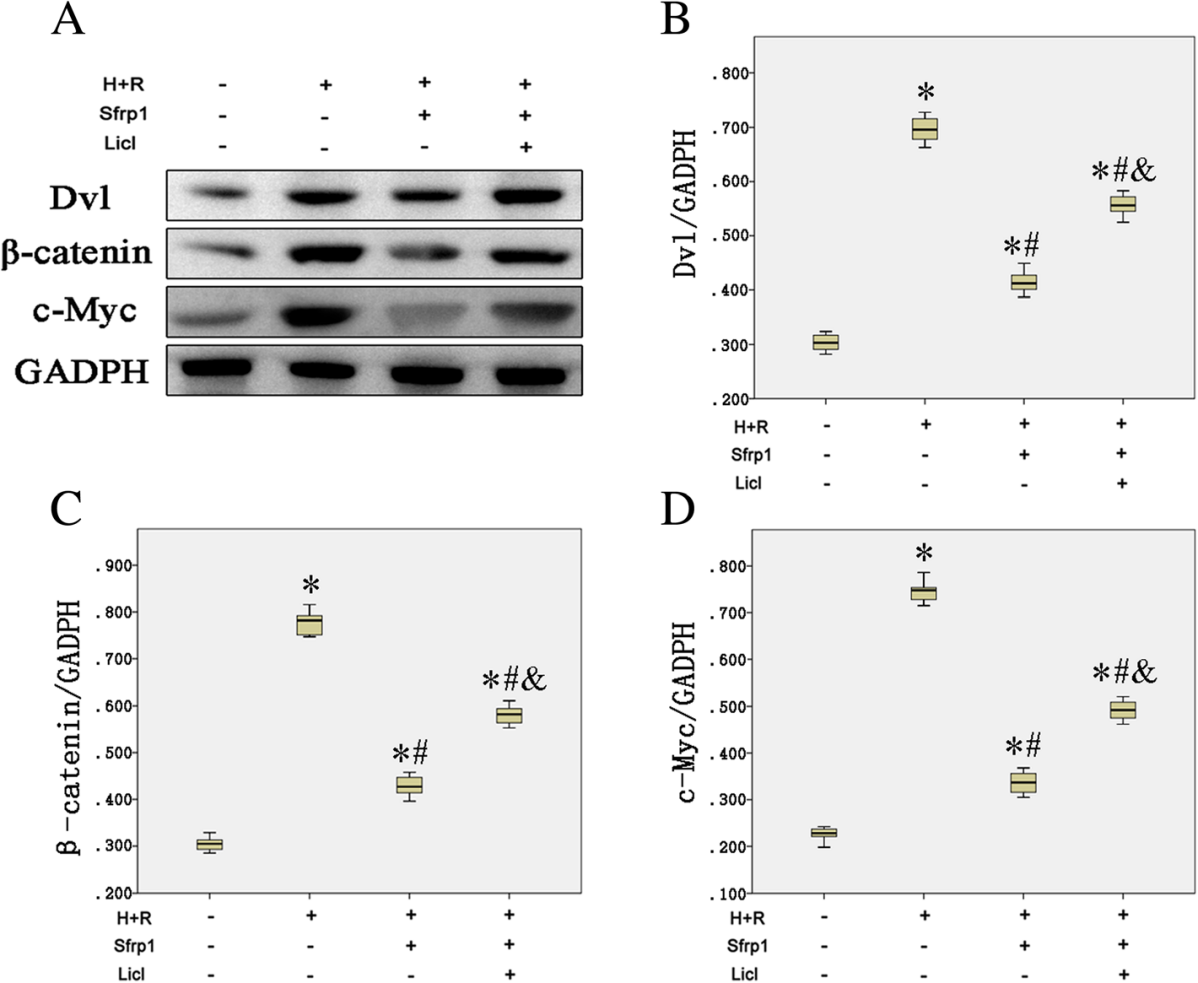
**a** Western blot detected the expression of Wnt signaling pathway key molecules. Hypoxia and reoxygenation (H+R) caused a marked increase in the protein levels of Dvl, β-catenin, and c-Myc. These changes were antagonized by Sfrp1-transfected cells before hypoxia and reoxygenation (Sfrp1+H+R); however, pre-treatment with the Wnt signaling pathway activator, Licl, increased the expression of Dvl, β-catenin, and c-Myc. **b** The median value of Dvl/GADPH with interquartile range (*box*) and range (whiskers). **c** The median value of β-catenin/GADPH with interquartile range (*box*) and range (whiskers). **d** The median value of c-Myc/GADPH with interquartile range (*box*) and range (whiskers). Post-hoc analyses of group differences were assessed with Kruskal-Wallis pairwise comparison. Statistical significance was set at the standard level (*p*<0.05), and the Bonferroni correction was applied in post-hoc analyses (α=0.05/6=0.0083). (Note:* vs. control *P*<0.001; # vs. H+R *P*<0.001; vs. Sfrp1+H+R *P*<0.001)

The above findings indicated that reactivation of the Wnt signaling pathway reduces the cardioprotective effects of Sfrp1, suggesting a close functional relationship. To determine whether or not AAV9-Sfrp1 inhibits the Wnt/frizzled pathway, Western blot analysis was performed in H9C2 cells to determine the level of expression of Dvl, β-catenin, and c-Myc, which were the main molecules involved in Wnt signaling pathway. As shown in Fig. 5, H+R caused a marked increase in the protein levels of Dvl, β-catenin, and c-Myc compared with control cells. AAV9-Sfrp1 transfection before H+R decreased the expression of Dvl, β-catenin, and c-Myc compared with the H+R only cells (*P* <0.001). Co-administration of Licl increased the expression of Dvl, β-catenin, and c-Myc.

## Discussion

Acknowledgement of the endogenous mechanisms that the heart relies on inadverse conditions represents a new focus in cardiologic research. In this setting, Sfrp1 can improve myocardial rescue from an ischemic insult by regulating the underlying mechanism and might provide a better therapeutic approach.

Sfrps possesses a cysteine-rich domain (CRD) that is similar to a homologous region on the frizzled receptor that binds Wnts [8, 13, 14], and are thought to bind and sequester Wnts away from active receptor complexes for inhibition of Wnt activity [15–17]. Previous observations demonstrate a role for Sfrps in the regulation of apoptosis. Some studies have indicated an anti-apoptotic role of Sfrp2 in mediating cellular resistance to UV, TNF, or ischemia-induced apoptosis in mammalian cell lines [18–21]. Sfrp1 and Sfrp2 are produced by long-term and ex vivo malignant glioma cells and are thought to act as survival and proliferation-promoting factors [22]. The current findings provide the first information that Sfrp1 also has a direct cytoprotective effect on cardiac muscle cells subjected in vitro to hypoxia and reoxygenation, increasing resistance to oxygen deprivation-induced apoptosis. This finding is in keeping with the observation that Sfrp1 protects cardiomyocytes against oxidative stress-induced apoptosis [9].

The Sfrp1-induced protection was observed in H9C2cells. In particular, our findings demonstrate that the protective action of Sfrp1 involves inhibition of the Wnt signaling pathway. The Wnt signaling pathway plays a crucial role in regulating numerous cellular processes, [23]. In the adult heart, the Wnt signaling pathway is quiescent under normal conditions [24, 25]. However, the Wnt signaling pathway is reactivated after injury and in various pathologic states or repair processes [23]. There is evidence indicating that the aberrant activation of the Wnt signaling pathway is related to apoptosis in several cell types [26, 27]. A previous study showed that conditional activation of the Wnt signaling pathway induces a marked increase in the frequency of apoptosis in hematopoietic stem/progenitor cells [28]. Expression of dominant stable beta-catenin causes significant apoptosis in human ovarian surface epithelial cells [29]. Similar results were also obtained in the human cell line of T/C28a [30]. Furthermore, knocking down the expression of Dvl-1 partially suppresses the activity of the Wnt signaling pathway and decreases the apoptotic rate, caspase-3 activity [31]. In the present experimental conditions, the expression of major molecules in the Wnt signaling pathway, such as Dvl, β-catenin, and c-myc were markedly increased in H+R-exposed H9C2cells. Sfrp1-transfected cells were capable of significantly inducing down-regulation of these molecules. This mechanism plays a major role in the cytoprotective action of Sfrp1 on cardiacmuscle cells. In fact, the capability of Sfrp1 to increase cell viability by reducing H+R-dependent oxidative stress and apoptosisis significantly hampered by co-administration of Licl, an activator for the Wnt signaling pathway [32]. The fact that Licl does not completely abolish the effects of Sfrp1 may suggest that multiple cytoprotective signaling pathways are operated by Sfrp1 in cardiac muscle cells, but the possible mechanisms remain to be elucidated.

Our study showed that Sfrp1 induces cytoprotection of H9C2 cells, which was exposed to H+R to mimic a reperfusion myocardial infarction model in vitro through inhibition of the Wnt signaling pathway in the cells, supporting the in vivo protective effect of Sfrp1 and the therapeutic potential for the ischemic heart. Thus, in H+R-induced injury, Wnt signaling plays a pathophysiologic role and antagonism of Wnt signaling may be important for cell protection.

## Conclusions

In conclusion, the present study determined that Sfrp1 directly protects H9C2 cells from H+R-induced reperfusion injury and apoptosis through inhibition of the Wnt signaling pathway; however, the present findings have been obtained on embryonic cardiac muscle cells, which may not behave similarly to adult cardiomyocytes of the heart in vivo. Due to the complicated nature of the in vivo environment, further in vivo studies will be conducted to confirm the cardioprotective effects of Sfrp1 on therapy of ischemic heart disease.

## Additional files

The gating strategy for flow cytometry to detect the apoptosis rate. Additional file 1: Figure S1. The gating strategy for flow cytometry to detect the apoptosis rate in the control group. (TIF 54 kb) Additional file 2: Figure S2. The gating strategy for flow cytometry to detect the apoptosis rate in the H+R group. (TIF 61 kb) Additional file 3: Figure S3. The gating strategy for flow cytometry to detect the apoptosis rate in the H+R+Sfrp1 group. (TIF 61 kb) Additional file 4: Figure S4. The gating strategy for flow cytometry to detect the apoptosis rate in the H+R+Sfrp1+Licl group. (TIF 63 kb)
