# Supplementary figures and images for: Secreted frizzled related protein 1 protects H9C2 cells from hypoxia/re-oxygenation injury by blocking the Wnt signaling pathway
Source: Lipids Health Dis. 2016 Apr 6;15:72. doi: 10.1186/s12944-016-0240-5 (PMC4822324; doi:10.1186/s12944-016-0240-5)

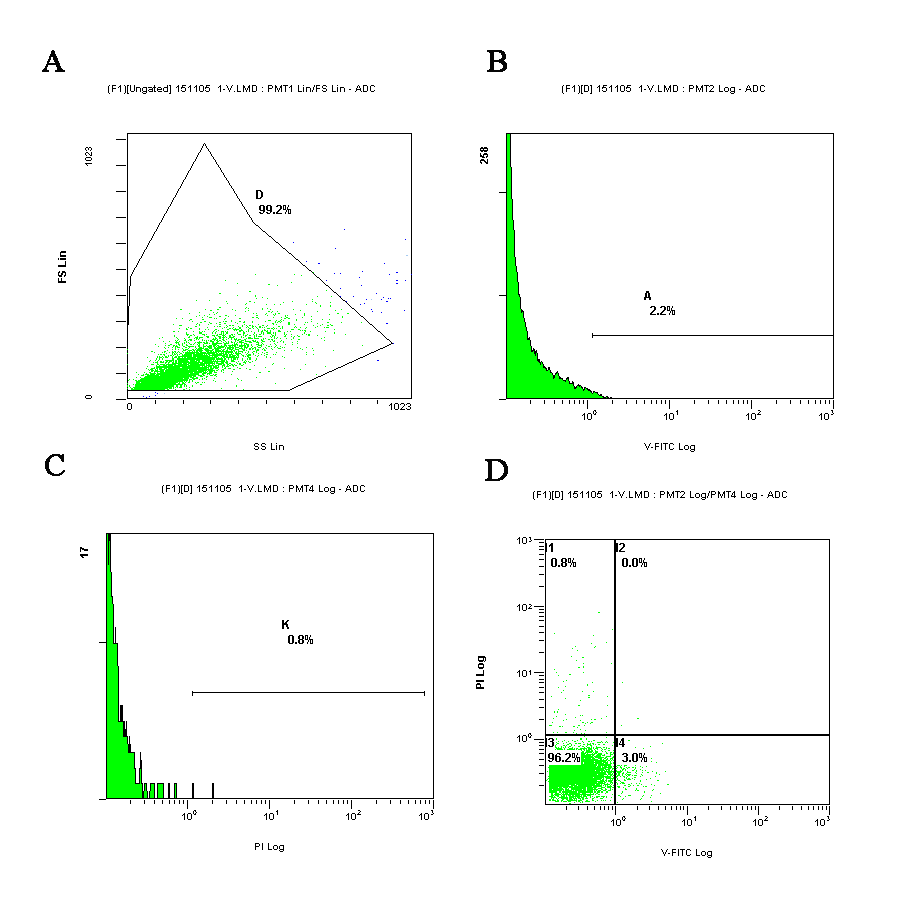

Supplement: Additional file 1: Figure S1. — The gating strategy for flow cytometry to detect the apoptosis rate in the control group. (TIF 54 kb) [file 12944_2016_240_MOESM1_ESM.tif]

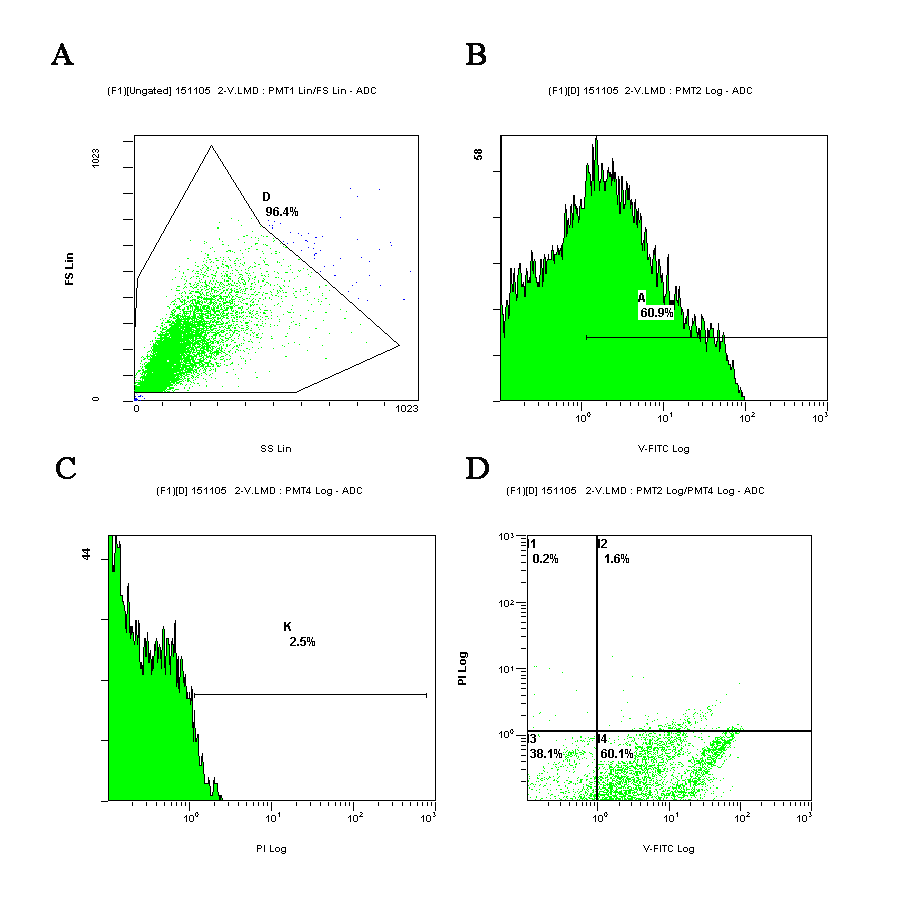

Supplement: Additional file 2: Figure S2. — The gating strategy for flow cytometry to detect the apoptosis rate in the H+R group. (TIF 61 kb) [file 12944_2016_240_MOESM2_ESM.tif]

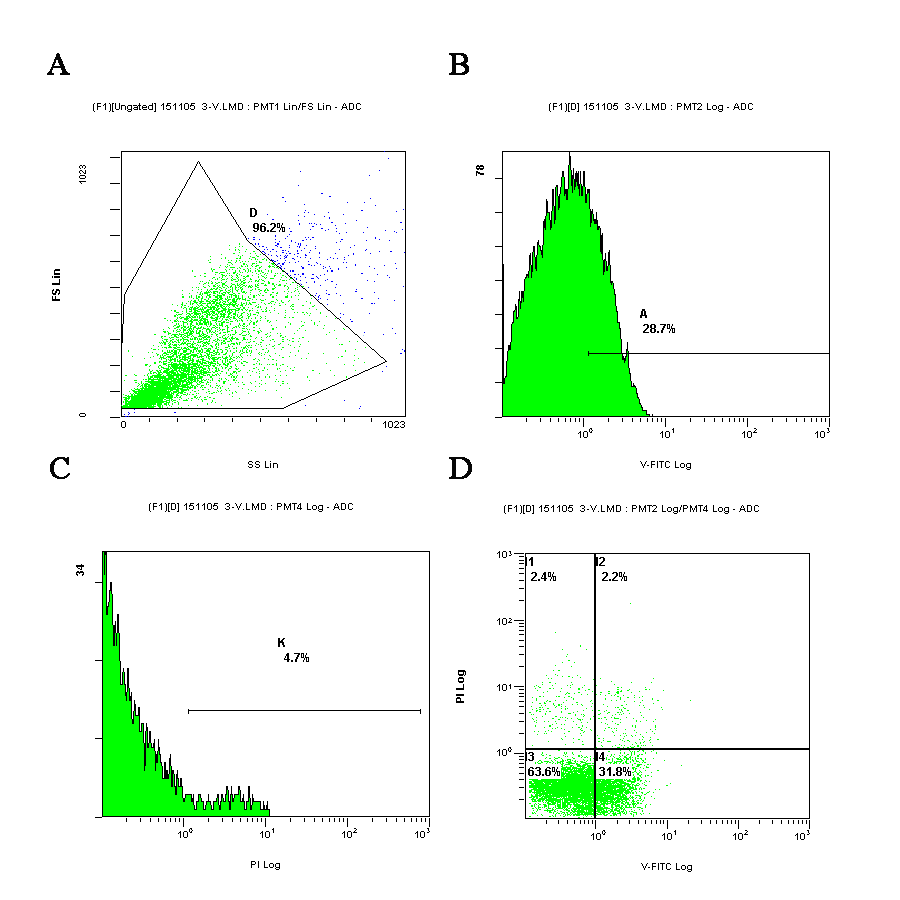

Supplement: Additional file 3: Figure S3. — The gating strategy for flow cytometry to detect the apoptosis rate in the H+R+Sfrp1 group. (TIF 61 kb) [file 12944_2016_240_MOESM3_ESM.tif]

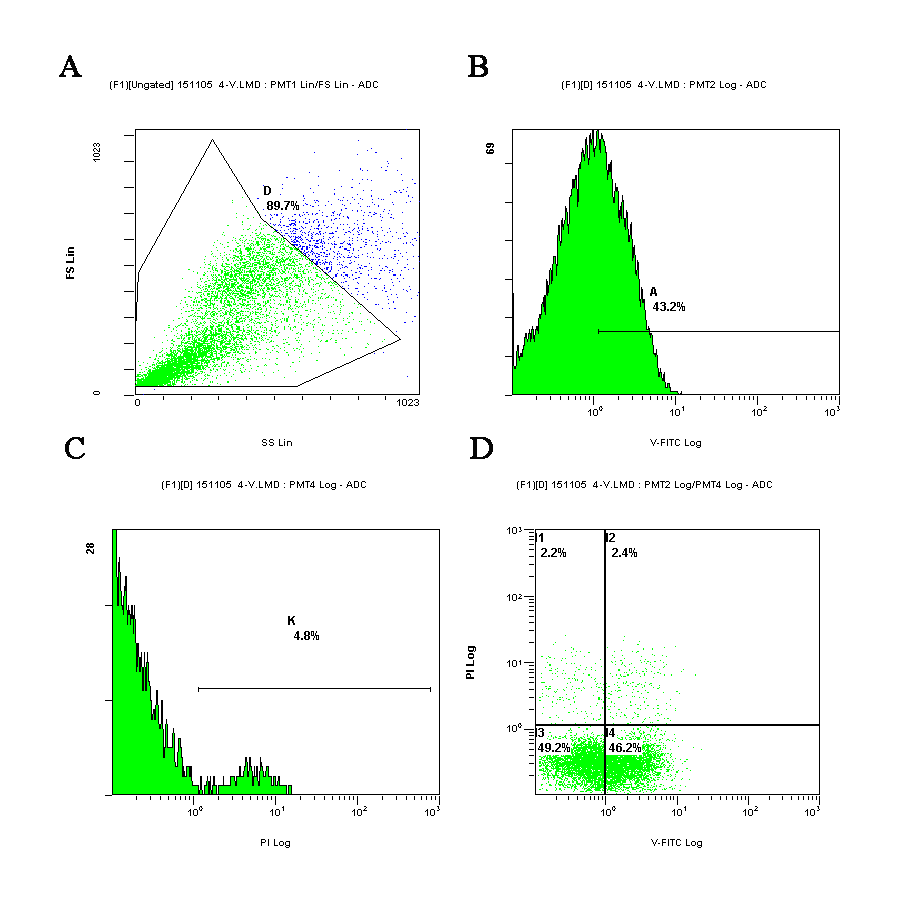

Supplement: Additional file 4: Figure S4. — The gating strategy for flow cytometry to detect the apoptosis rate in the H+R+Sfrp1+Licl group. (TIF 63 kb) [file 12944_2016_240_MOESM4_ESM.tif]
